# An Intelligence EEG Signal Recognition Method via Noise Insensitive TSK Fuzzy System Based on Interclass Competitive Learning

**DOI:** 10.3389/fnins.2020.00837

**Published:** 2020-09-04

**Authors:** Tongguang Ni, Xiaoqing Gu, Cong Zhang

**Affiliations:** School of Computer Science and Artificial Intelligence, Changzhou University, Changzhou, China

**Keywords:** noise insensitive, TSK fuzzy system, Bayesian framework, possibilistic clustering, Ho–Kashyap procedure, asymmetric expectile term

## Abstract

Epilepsy is an abnormal function disease of movement, consciousness, and nerve caused by abnormal discharge of brain neurons in the brain. EEG is currently a very important tool in the process of epilepsy research. In this paper, a novel noise-insensitive Takagi–Sugeno–Kang (TSK) fuzzy system based on interclass competitive learning is proposed for EEG signal recognition. First, a possibilistic clustering in Bayesian framework with interclass competitive learning called PCB-ICL is presented to determine antecedent parameters of fuzzy rules. Inherited by the possibilistic *c*-means clustering, PCB-ICL is noise insensitive. PCB-ICL learns cluster centers of different classes in a competitive relationship. The obtained clustering centers are attracted by the samples of the same class and also excluded by the samples of other classes and pushed away from the heterogeneous data. PCB-ICL uses the Metropolis–Hastings method to obtain the optimal clustering results in an alternating iterative strategy. Thus, the learned antecedent parameters have high interpretability. To further promote the noise insensitivity of rules, the asymmetric expectile term and Ho–Kashyap procedure are adopted to learn the consequent parameters of rules. Based on the above ideas, a TSK fuzzy system is proposed and is called PCB-ICL-TSK. Comprehensive experiments on real-world EEG data reveal that the proposed fuzzy system achieves the robust and effective performance for EEG signal recognition.

## Introduction

Epilepsy occurs randomly and may occur multiple times in a day. In the case of epileptic seizures, the patients have a sudden physical convulsions and loss of consciousness, which bring great physical and psychological pain to patients (Ahmadlou and Adeli, 2011; Gummadavelli et al., 2018; Cury et al., 2019). Seizures will lead to brain cell death, affect brain function, and even threaten patients' lives in serious cases. The incidence of epilepsy is high, and the age range is very wide, including children, adolescents, and the elderly, but the incidence of children and adolescents is the highest. Both men and women are likely to have the disease, and men are more likely to have this disease than women. As an important clinical means of monitoring and diagnosing epilepsy, EEG provides a more rapid and stable low-cost and non-invasive technology in monitoring the brain activity of the cerebral cortex. It provides information that other physiological methods cannot provide. The specific waveforms such as spike, sharp, and complex wave can be reflected by EEG. Therefore, the prevention and treatment of epilepsy research for epilepsy patients is of great significance. In the process of diagnosis and treatment of epilepsy, EEG plays an irreplaceable role. Doctors usually judge the condition of patients by observing their EEG.

The traditional way to judge the EEG signal is not only inefficient, but also because of the difference of experts' subjective experience, the automatic detection of EEG signal is still one of the hot issues in biomedical research (Jiang et al., 2017a; Martinez-Vargas et al., 2017; Li et al., 2019). An automatic epilepsy detection method can help doctors improve the accuracy of epilepsy diagnosis and also greatly save time. The research of automatic epilepsy detection is of great value to the prevention, diagnosis, and treatment of epilepsy. At present, epilepsy can be detected by machine learning and data mining. Firstly, the effective feature information is extracted from EEG and preprocessed for data analysis; secondly, the preprocessed EEG data are sent to the classifier for analysis and detection of epileptic and non-epileptic EEG data. In the above implementation process, the key research is to design an effective prediction and discrimination method that can be applied to normal EEG signal and epileptic EEG signal. Many effective methods have been successfully applied to automatic epilepsy detection system, including extreme learning machine (ELM), artificial neural network, Bayesian linear discriminant analysis, support vector machine (SVM), and fuzzy system (Kabir and Zhang, 2016; Qi et al., 2017; Akhavan and Moradi, 2018; Truong et al., 2018; Hossain et al., 2019; Liu et al., 2019; Sreej and Samanta, 2019; Xia et al., 2020). The fuzzy system is a model constructed to deal with the thinking, analysis, reasoning, and decision-making processes in production and practice. It can directly translate natural language into computer language. Due to its ability to process uncertain and ambiguous information, it has a high degree of interpretability and strong learning ability (Juang et al., 2007; Gu et al., 2017a; Jiang et al., 2017b,c; Gu and Wang, 2018). However, the traditional fuzzy system has poor robustness and anti-interference ability, and the classification accuracy is not high in data noise scenarios. But in real life, the classification of noise data is widely used. For example, in actual application scenarios, due to differences in machine advices or scanning technology, such as different rotation angles and noise, the quality of medical images may vary greatly (Siuly and Li, 2015; Hussein et al., 2019; Razzak et al., 2019).

**Figure 1:**
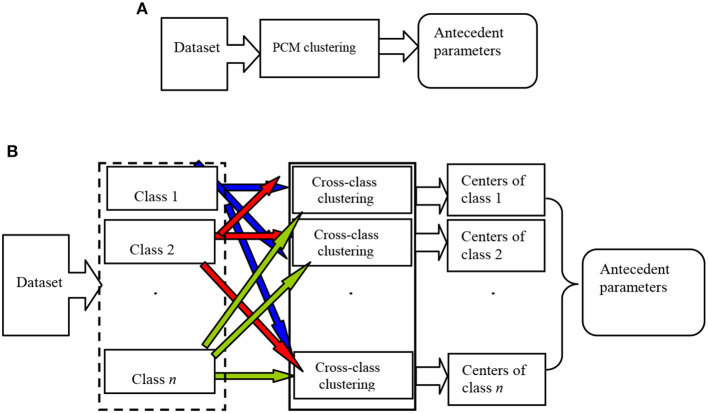
Principle of antecedent parameter learning using PCB-ICL clustering. **(A)** The principle of antecedent parameter learning using PCM clustering. **(B)** The principle of antecedent parameter learning using PCB-ICL clustering.

Based on key technology of fuzzy system modeling, this paper proposes a novel noise-insensitive Takagi–Sugeno–Kang (TSK) fuzzy system. How to determine the antecedent and consequent parameters is the key to modeling the noise-insensitive fuzzy system (Takagi and Sugeno, 1985; Jiang et al., 2015). For the antecedent part of fuzzy rules, clustering is one kind of a commonly used strategy, such as fuzzy *c*-means (FCM) clustering (Bezdek et al., 1984), fuzzy (*c* + *p*) clustering (Leski, 2015), Bayesian fuzzy clustering (BFC) (Glenn et al., 2015), and possibilistic *c*-means (PCM) clustering (Krishnapuram and Keller, 1993). However, FCM, fuzzy (*c* + *p*), and BFC are sensitive to noise and will lead to unsatisfactory partition in noisy scenarios. PCM inherits the practicability and flexibility of fuzzy clustering and greatly enhances the clustering performance of data with noise or outliers. However, the unsupervised nature of PCM makes it unable to use the class label information of samples, which easily causes the insufficient fuzzy space partition, thus further affecting the learning of antecedent parameters of fuzzy rules. The principle of antecedent parameter learning using PCM clustering is shown in Figure 1A. PCM clustering is directly used on whole datasets or on samples in each class, and then the antecedent parameters are learned using the obtained clustering results. Then the data samples are simply divided into several clusters, without fully taking advantage of the geometry of data and the label information of samples. In this case, in the data overlapping regions, the distance between clustering centers may be too small or the centers may overlap.

In this paper, we first propose a noise-insensitive possibilistic clustering in Bayesian framework with interclass competitive learning called PCB-ICL. Inherited by PCB, PCB-ICL is noise insensitive; meanwhile, different classes of cluster centers will produce a competitive relationship during the learning process. That is, in the sample overlapping area, the clustering centers are attracted by the samples of the same class and also excluded by the samples of other classes and pushed away from the heterogeneous data. The principle of antecedent parameter learning using PCB-ICL clustering is shown in Figure 1B. PCB-ICL integrates the competitive learning mechanism of clustering centers among different classes in the Bayesian framework. PCB-ICL considers the structure information of samples in the clustering procedure and realizes the competition between clustering centers among different classes. We obtain the antecedent part of fuzzy rules by performing PCB-ICL alternatively on each class samples. Then, a Ho–Kashyap procedure (Leski, 2003) with an asymmetric expectile term (Huang et al., 2014a,b) is adopted to estimate the consequent parameters of fuzzy rules. Due to the statistical characteristics of the asymmetric expectile term, it is insensitive to noise; so the asymmetric expectile term is used to measure the misclassification error. Based on the above idea, the TSK fuzzy system called PCB-ICL-TSK is developed, which learns antecedent parameters by PCB-ICL clustering and consequent parameters by the Ho–Kashyap procedure with an asymmetric expectile term. We apply the proposed algorithm on the Bonn EEG dataset, and the experimental results on several noisy classification tasks demonstrate that PCB-ICL-TSK can achieve satisfactory performance in EEG signal classification. The novelty of our study is as follows. (1) Both the PCB-ICL and Ho–Kashyap procedure with an asymmetric expectile term are insensitive to noise; thus, the obtained antecedent and consequent parameters are noise insensitive. (2) With the Bayesian framework, the clustering results of PCB-ICL are globally optimal. In addition, the competitive relationship strategy between cluster centers enhances the interpretability of the antecedents of fuzzy rules. (3) The experiments on real-word EEG datasets confirm the effectiveness of PCB-ICL-TSK.

The detailed chapters are arranged as follows. Section Backgrounds introduces the TSK fuzzy system and PCM clustering. Section Possibilistic Clustering in Bayesian With Interclass Competitive Learning explores PCB-ICL clustering. Section Noise-Insensitive TSK Fuzzy System via Interclass Competitive Learning explores the noise-insensitive TSK fuzzy system PCB-ICL-TSK. Section Experiment is experiments on noisy EEG data. Section Conclusion is the conclusion.

## Backgrounds

### Dataset

**Figure 2:**
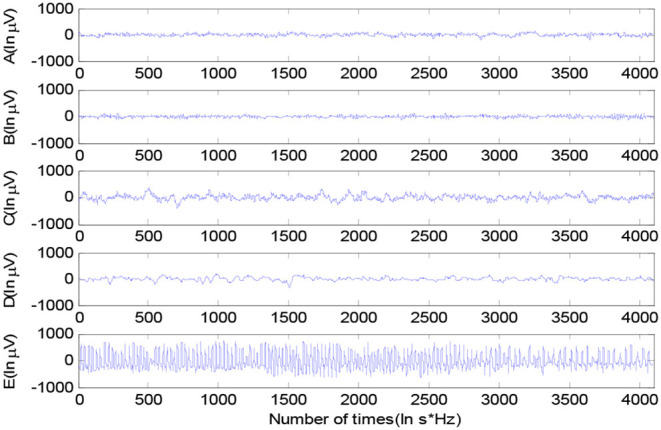
The epileptic EEG signals in groups A to E.

The epileptic EEG in the experiment is the Bonn dataset from Bonn University, Germany (Tzallas et al., 2009). The Bonn EEG dataset consists of five groups of data, namely, A to E, shown in Figure 2. Each group of data contains 100 EEG signal segments of 23.6 s, which were selected from continuous single-channel EEG recordings. The EEG signals were recorded under different conditions with five patients and five healthy volunteers. The basic information of groups A–E is shown in Table 1.

**Table 1:** The basic information of EEG data groups of A–E.

	**Group**	**Description**
Healthy volunteers	A	EEG signals of healthy volunteers in an awakened state with eyes open
	B	EEG signals of healthy volunteers in an awakened state with eyes closed
Patients	C	EEG signals of patients in hippocampal formation of the opposite hemisphere of the brain
	D	EEG signals of patients in the epileptogenic zone during periodic lulls
	E	EEG signals of patients during seizure activity

### TSK Fuzzy System

The most commonly used rule in the zero-order TSK fuzzy system can be represented by

Rule *R*_*k*_: IF *x*_1_ is *A*_*k*__,1_ and *x*_2_ is *A*_*k*__,2_ and … and *x*_*d*_ is *A*_*k*_,_*d*_,

(1) $$\begin{array}{l} then\;f_{k}\left(\text{x}\right)=P_{k,0},\left(k=1,2,\ldots ,K\right) \end{array}$$

where *x*_1_, *x*_2_, …, *x*_*d*_ are input variables, *A*_*k*_,_*i*_ is a fuzzy subset, and *K* is the number of fuzzy rules. For an input vector **x**, the output of the corresponding TSK fuzzy system is represented by

(2) $$\begin{array}{l} {y\;}_{output}=\frac{\overset{K}{\underset{k=1}{\sum }}\mu_{k}\left(\text{x}\right)p_{k,0}^{\;}}{\overset{K}{\underset{k=1}{\sum }}\mu_{k}\left(\text{x}\right)}=\overset{K}{\underset{k=1}{\sum }}{\tilde{\mu }}_{k}\left(\text{x}\right)p_{k,0}^{\;}, \end{array}$$

where the fuzzy membership μ_*k*_(**x**) and the normalized fuzzy membership ${\tilde{\mu }}_{k}\left(\text{x}\right)$ is

(3) $$\begin{array}{l} \mu_{k}\left(\text{x}\right)=\prod_{i=1}^{d}\mu_{A_{k,i}^{\;}}\left(x_{i}\right), \end{array}$$

(4) $$\begin{array}{l} {\tilde{\mu }}_{k}\left(\text{x}\right)=\frac{\mu_{k}\left(\text{x}\right)}{\overset{K}{\underset{k^{{\;}^{\prime }}=1}{\sum }}\mu_{k^{{\;}^{\prime }}}\left(\text{x}\right)}. \end{array}$$

For the sample **x**_*i*_, we can rewrite it by

(5) $$\begin{array}{l} d\left({\text{x}}_{i}\right)={\left[{\tilde{\mu }}_{1}\left({\text{x}}_{i}\right),{\tilde{\mu }}_{2}\left({\text{x}}_{i}\right),\ldots ,{\tilde{\mu }}_{K}\left({\text{x}}_{i}\right)\right]}^{T}, \end{array}$$

Generally, antecedent and consequent parameters of rules are determined separately. A popular way to estimate antecedent parameters is to use a certain fuzzy clustering method (Takagi and Sugeno, 1985; Gu et al., 2017b; Salgado et al., 2017). Then $\mu_{A_{k,i}^{\;}}\left(x_{i}\right)$ can be computed by

(6) $$\begin{array}{l} \mu_{A_{k,i}^{\;}}\left(x_{i}\right)=\text{exp}\left(-\frac{{\left(x_{i}-y_{k,i}^{\;}\right)}^{2}}{2\delta_{k,i}^{\;}}\right), \end{array}$$

where the width parameter δ_*k*_,_*i*_ can be obtained by

(7) $$\begin{array}{l} \delta_{k,i}^{\;}=\frac{h\cdot \overset{N}{\underset{j=1}{\sum }}u_{k,j}{\left(x_{ji}-y_{k,i}^{\;}\right)}^{2}}{\overset{N}{\underset{j=1}{\sum }}u_{k,j}}, \end{array}$$

where *h* is the scale parameter and *u*_*k*_,_*j*_ is the fuzzy membership of the *j*th input sample **x**_*j*_ belonging to the *k*th cluster.

Then the learning of consequent parameters can be represented by

(8) $$\begin{array}{l} \underset{\text{p}}{\min}\;\overset{N}{\underset{i=1}{\sum }}|l_{i}d{\left({\text{x}}_{i}^{\;}\right)}^{T}\text{p}-1| \end{array}$$

Using the least square solution to minimize the squared loss, Equation (8) can be written by

(9) $$\begin{array}{l} \underset{\text{p}}{\min}\;J\left(\text{p}\right)={\left(\text{D}\text{p}-1_{N\times 1}\right)}^{T}\text{H}\left(\text{D}\text{p}-1_{N\times 1}\right)+\tau {\text{p}}^{T}\text{p}, \end{array}$$

where **D** = [*l*_1_*d*(**x**_1_)^*T*^, …, *l*_*N*_*d*(**x**_*N*_)^*T*^]^*T*^, the matrix **H** = diag(*h*_1_, *h*_2_, …, *h*_*N*_), *h*_*i*_ = 1/|*l*_*i*_*d*(**x**_*i*_)^*T*^***p***−1| for *l*_*i*_*d*(**x**_*i*_)^*T*^***p***−1 < 0, and *h*_*i*_ = 0 otherwise. τ is the regularization parameter. Using the Ho–Kashyap iterative method (Leski, 2003), **p** can be computed by

(10) $$\begin{array}{l} \text{p}={\left({\text{D}}^{T}\text{H}\text{D}+\tau \text{I}\right)}^{-1}{\text{D}}^{T}\text{H}1, \end{array}$$

where **I** is the identify matrix.

### PCM Clustering

PCM clustering is a probability clustering based on FCM. Based on the framework of possibility theory, PCM not only takes into account the general criteria of clustering with the minimum distance within one class and the maximum distance between classes but also emphasizes the principle of the maximum membership value to avoid ordinary solution problems. The objective function of PCM is

(11) $$\begin{array}{l} \underset{\text{U},\text{Y}}{\min}\;\overset{N}{\underset{n=1}{\sum }}\overset{C}{\underset{c=1}{\sum }}u_{nc}^{m}{\left({\text{x}}_{n}-{\text{y}}_{c}\right)}^{2}+\overset{N}{\underset{n=1}{\sum }}\overset{C}{\underset{c=1}{\sum }}\eta_{c}{\left(1-u_{nc}^{\;}\right)}^{m},\\ \;s.t.\;u_{nc}^{\;}\in \left[0,1\right],\forall n,c \end{array}$$

The closed solution of **U** and **Y** can be obtained by minimizing the objective function with respect to *u*_*nc*_ and **y**_*c*_ by.

(12) $$\begin{array}{lll} {\text{y}}_{c} & = & \frac{\overset{N}{\underset{n=1}{\sum }}u_{nc}^{m}{\text{x}}_{n}}{\overset{N}{\underset{n=1}{\sum }}u_{nc}^{m}} \end{array}$$

(13) $$\begin{array}{lll} u_{nc}^{\;} & = & \frac{1}{1+{\left(\frac{{\left({\text{x}}_{n}-{\text{y}}_{c}\right)}^{2}}{\eta_{c}}\right)}^{\frac{1}{m-1}}} \end{array}$$

## Possibilistic Clustering in Bayesian with Interclass Competitive Learning

### Objective Function

A clustering method implements data partition with some certain degree of similarity. In the clustering process, the samples of one class will have a repulsive effect on the clustering center of other classes, especially in the overlapping regions of different classes of samples; the greater the overlap density, the greater the repulsive force. In these sample overlapping regions, clustering centers of different classes form the competitive learning relationship. On the one hand, the clustering centers are attracted by samples of this class; on the other hand, the clustering centers are excluded by different classes of samples and far away from the overlapping region. In this paper, this idea is embedded into PCM clustering. Based on the Bayesian framework, we propose the possibilistic clustering in Bayesian with interclass competitive learning.

Suppose a given binary classification dataset $\text{X}={\left\{{\text{x}}_{n},l_{n}\right\}}_{n=1}^{N}$, in which ${\text{X}}_{1}={\left\{{\text{x}}_{n},l_{n}\right\}}_{n=1}^{N_{1}}$ and ${\text{X}}_{2}={\left\{{\text{x}}_{n},l_{n}\right\}}_{n=N_{1}+1}^{N}$ represent two class samples and *l*_*n*_ ∈ {+1, −1} is the class label of the *n*th sample. Let the cluster number of one class samples be *C*_1_ and the cluster centers of the other class **Z** be priorly known $\text{Z}={\left[{\text{z}}_{1}\text{,}{\text{z}}_{2},\ldots ,{\text{z}}_{{\text{c}}_{2}}\right]}^{T}$, where the cluster number is *C*_2_. We suppose data **X** follows the normal distribution, and each sample **x**_*i*_ has an independent probability distribution. The maximum posterior estimation of data and parameters in **X**_1_ is expressed by

(14) $$\begin{array}{l} p\left({\text{X}}_{1},\text{U},\text{Y}\right)=p\left({\text{X}}_{1}|\text{U},\text{Y}\right)p\left(\text{U}|\text{Y}\right)p\left(\text{Y}\right)\\ \;\propto exp\left\{-\frac{1}{2}\left(\overset{N_{1}}{\underset{n=1}{\sum }}\overset{C_{1}}{\underset{c=1}{\sum }}u_{nc}^{m}{\left\|{\text{x}}_{n}-{\text{y}}_{c}\right\|}^{2}+\overset{N_{1}}{\underset{n=1}{\sum }}\overset{C_{1}+C_{2}}{\underset{c=C_{1}+1}{\sum }}u_{nc}^{m}{\left\|{\text{x}}_{n}-{\text{z}}_{c}\right\|}^{2}\right)\right\}\times \\ \;\left[\prod_{n=1}^{N_{1}}\prod_{c=1}^{C_{1}+C_{2}}\text{exp}(-\frac{1}{2}\eta_{c}{\left(1-u_{nc}\right)}^{m})\times \text{exp}\left\{-\frac{1}{2}\sum_{c=1}^{C_{1}}{\text{(}y_{c}-\mu_{y})}^{T}\sum_{y}^{-1}\text{(}y_{c}-\mu_{y})\right\}\right], \end{array}$$

where $\text{Y}={\left[{\text{y}}_{1}\text{,}{\text{y}}_{2},\ldots ,{\text{y}}_{c_{1}}\right]}^{T}$ is the unknown cluster center matrix of one class sample. By taking the logarithm of Equation (14), the objective function of PCB-ICL method can be obtained as

(15) $$\begin{array}{l} J\left({\text{X}}_{1},\text{U},\text{Y}\right)=\overset{N_{1}}{\underset{n=1}{\sum }}\overset{C_{1}}{\underset{c=1}{\sum }}u_{nc}^{m}{\left\|{\text{x}}_{n}-{\text{y}}_{c}\right\|}^{2}+\overset{N_{1}}{\underset{n=1}{\sum }}\overset{C_{1}+C_{2}}{\underset{c=C_{1}+1}{\sum }}u_{nc}^{m}{\left\|{\text{x}}_{n}-{\text{z}}_{c}\right\|}^{2}\\ \;+\overset{N_{1}}{\underset{n=1}{\sum }}\overset{C_{1}+C_{2}}{\underset{c=1}{\sum }}\eta_{c}{\left(1-u_{nc}^{\;}\right)}^{m}\\ \;+\sum_{c=1}^{C_{1}}{\text{(}y_{c}-\mu_{y})}^{T}\sum_{y}^{-1}\text{(}y_{c}-\mu_{y}). \end{array}$$

From Equations (14) and (15), we can see that (1) the PCB-ICL method shows the competition relationship between clustering centers of different classes. Different from the traditional PCM clustering method, PCB-ICL not only considers the label information of samples but also considers the competition relationship between clustering centers, as shown in the first two items. On the premise that the clustering centers of the other class are priorly known, the clustering centers of the current class will inevitably have a competition relationship with these known clustering centers in the overlapping region. (2) Due to simultaneously utilizing the global distribution structure and the discrimination information of the samples, the obtained antecedent part of fuzzy rules by PCB-ICL can realize the clarity of fuzzy space partition and enhance the interpretability of the fuzzy rules.

### Parameter Learning

To obtain the optimal fuzzy partition matrix **U**, the PCB-ICL method uses the Metropolis–Hastings method (Chib and Greenberg, 1995; Elvira et al., 2017) to construct a Markov chain to make *p*(**U**|**X**_1_, **Y**) stable. The conditional distribution *p*(**U**|**X**_1_, **Y**) is proportional to the joint distribution *p*(**X**_1_, **U**, **Y**) when the sample and clustering center are known and also is proportional to the conditional distribution *p*(**U**|**X**_1_, **Y**). Therefore, we only need compute *p*(**x**_*n*_, **u**_*n*_|**Y**) of the sample **x**_*n*_:

(16) $$\begin{array}{l} p\left({\text{x}}_{n},{\text{u}}_{n}|\text{Y}\right)=p\left({\text{x}}_{n}|{\text{u}}_{n},\text{Y}\right)p\left({\text{u}}_{n}|\text{Y}\right)\\ \;\propto exp\left\{-\frac{1}{2}\left(\overset{C_{1}}{\underset{c=1}{\sum }}u_{nc}^{m}{\left\|{\text{x}}_{n}-{\text{y}}_{c}\right\|}^{2}+\overset{C_{1}+C_{2}}{\underset{c=C_{1}+1}{\sum }}u_{nc}^{m}{\left\|{\text{x}}_{n}-{\text{z}}_{c}\right\|}^{2}\right)\right\}\\ \;\times \overset{C_{1}+C_{2}}{\underset{c=1}{\prod }}\exp\left(-\frac{1}{2}\eta_{c}{\left(1-u_{nc}^{\;}\right)}^{m}\right). \end{array}$$

Thus, the process of the *i*th iteration of the Markov chain is

1) Generate a new state ${\text{u}}_{n}^{+}$ of **u**_*n*_ with a uniform distribution as
  (17) $$u_{n}^{+}\;\sim \text{ Uniform}(0,1\text{),}\forall n$$
2) The newly generated membership ${\text{u}}_{n}^{+}$ is accepted by the probability *a*_***u***_ as
  (18) $$a_{u}=\min\left\{1,\frac{p(x_{\text{n}},u_{n}^{+}|Y)}{p(x_{\text{n}},u_{n}|Y)}\right\}$$
  Then accepting *a*_***u***_ as the current state with probability **u**_*n*_,
  (19) $$\begin{array}{l} {\text{u}}_{n}^{\;}=\{\begin{array}{c} {\text{u}}_{n}^{+},\;\mu \leq \alpha_{\text{u}}\\ {\text{u}}_{n},\;\mu >\alpha_{\text{u}} \end{array} \end{array}$$
  where μ is a random number in [0, 1]. The distribution of the new state ${\text{u}}_{n}^{+}$ obtained by sampling is independent of the current sample, and the state ${\text{u}}_{n}^{+}/{\text{u}}_{n}^{\;}$ is independent, so *a*_**u**_ does not need Hasting correction.
3) Compare $p({\text{x}}_{n}\text{,}{\text{u}}_{n}^{+}\;|\;{\text{Y}}_{\;}^{*}\text{)}$ and $p\left({\text{x}}_{n},{\text{u}}_{n}^{*}\;|\;{\text{Y}}_{\;}^{*}\right)$, where Y^*^ and ${\text{u}}_{n}^{*}$ are the optimal values of **Y** and **u**_*n*_. If, $p(x_{n}\text{,}u_{n}^{+}\text{|}Y^{*}\text{) >}p\left(x_{n},u_{n}^{*}\text{|}Y^{*}\right)$ ${\text{u}}_{n}^{+}$ is replaced by ${\text{u}}_{n}^{*}$.
  When the matrix **U** is fixed, we use Metropolis–Hastings to sample the conditional distribution *p*(**Y**|**X**, **U**). In this case, *p*(**Y**|**X**, **U**) is proportional to the joint distribution *p*(**X**, **U**, **Y**). We estimate **y**_*c*_ by using the Gaussian distribution as
  (20) $$\begin{array}{l} {\text{y}}_{c}^{+}\sim N\left({\text{y}}_{c},\frac{1}{\sigma }\sum_{y}\right) \end{array}$$

where ${\text{y}}_{c}^{+}$ centers on the current value **y**_*c*_. σ is a positive number and is used to control the compactness of cluster centers. In the experiment, we empirically set σ to 10.

For the newly generated ${\text{y}}_{c}^{+}$, it is independent of other clustering centers. Then the conditional distribution *p*(**X**, **y**_*c*_|**U**) is represented by

(21) $$\begin{array}{l} p\left(\text{X},{\text{y}}_{c}|\text{U}\right)=p\left(\text{X}\;|\;\text{U},{\text{y}}_{c}\right)p\left({\text{y}}_{c}\right)\\ \;\propto \exp\left\{-\frac{1}{2}\overset{N_{1}}{\underset{n=1}{\sum }}u_{nc}^{m}{\left\|{\text{x}}_{n}-{\text{y}}_{c}\right\|}^{2}\right\}\\ \;\times \exp\left\{-\frac{1}{2}{\text{(}y_{c}-\mu_{y})}^{T}\sum_{y}^{-1}\text{(}y_{c}-\mu_{y})\right\}. \end{array}$$

Similarly, the newly generated membership ${\text{y}}_{c}^{+}$ is accepted by the probability *a*_**y**_ as

(22) $$\begin{array}{l} a_{\text{y}}=\min\left\{1,\frac{p\left(\text{X},{\text{y}}_{c}^{+}|\text{U}\right)}{p\left(\text{X},{\text{y}}_{c}|\text{U}\right)}\right\} \end{array}$$

Since the Gaussian distribution is symmetric, *a*_**y**_ does not need Hasting correction.

Finally, we compute *p*(**X**, U^*^, Y^*^) using Equation (15) and compare it with the current *p*(**X**, **U**, **Y**). If *p*(**X**, **U**, **Y**) > *p*(**X**, U^*^, Y^*^), the {**U**, **Y**} is replaced by {U^*^, Y^*^}.

Based on the above analysis, we give the procedure of the PCB-ICL method in Algorithm 1.

**Algorithm 1:** PCB-ICL method.

**Input:** Dataset X_1_ of one class, the number of clustering *C*, priorly known clustering center matrix **Z** of the other class;
**Output:** Fuzzy partition matrix U* and clustering center matrix Y*.
Step 1 Initiate ${\text{u}}_{n}^{+}\;\sim \;Uniform\left(0,1\right),\forall n\;$; Step 2 Initiate ${\text{y}}_{c}^{+}\sim N\left({\text{y}}_{c},\frac{1}{\sigma }\sum_{y}\right)$, ∀*c*; Step 3 Set ${\text{u}}_{n}^{*}$= ${\text{u}}_{n}^{+}$, ${\text{y}}_{c}^{*}={\text{y}}_{c}^{+}$; For *iter* = 1, 2, …, *N_*iter*_* For *n* = 1, 2, …, *N* Step 4 Sample ${\text{u}}_{n}^{+}$ using Equation (17) and accept it as **u***_*n*_* using Equations (18) and (19); Step 5 If $p\left({\text{x}}_{n},{\text{u}}_{n}^{+}|{\text{Y}}^{*}\right)>p\left({\text{x}}_{n},{\text{u}}_{n}^{*}|{\text{Y}}^{*}\right)$, then ${\text{u}}_{n}^{*}$= ${\text{u}}_{n}^{+}$; Endfor For *c* = 1, 2, …, *C* Step 6 Sample ${\text{y}}_{c}^{+}$ using Equation (20) and accept it as ${\text{y}}_{c}^{*}$ using Equations (21) and (22); Step 7 If $p(\text{X},{\text{y}}_{c}^{+}|{\text{U}}^{*}\text{)}$ > $p(\text{X},{\text{y}}_{c}^{*}|{\text{U}}^{*}\text{)}$, then ${\text{y}}_{c}^{*}\leftarrow {\text{y}}_{c}^{+}$; Endfor Step 8 If *p*(**X**, U*, Y*) > *p*(**X**, **U**, **Y**), then U*← **U**, Y*← **Y**; Endfor.

## Noise-Insensitive TSK Fuzzy System via Interclass Competitive Learning

### Antecedent Parameter Learning in PCB-ICL-TSK

In this section, we compute the antecedent parameters in PCB-ICL-TSK. The premise of PCB-ICL clustering in Algorithm 1 is that the clustering centers of other class are priorly known, which is obviously not feasible in practical application. To perform the fuzzy partition on the whole data set, we take the strategy of an alternating cycle to perform Algorithm 1 on different classes. In this case, the clustering results of one class influence the ones of the other class. Taking binary classification as an example, we perform Algorithm 1 on positive class **X**_1_ and negative class **X**_2_ alternately. The detailed fuzzy partition of the whole data is shown in Algorithm 2.

**Algorithm 2:** Fuzzy partition on the whole data.

**Input:** Two class samples **X**_1_ and **X**_2_, the numbers of clustering *C*_1_ and *C*_2_ in two classes;
**Output:** Fuzzy partition matrix **U**^(1)*^, **U**^(2)*^ and clustering center matrix **Y**^(1)*^, **Y**^(2)*^.
Step 1 Initiate ${\text{u}}_{n}^{\left(1\right)}\;\left({\text{u}}_{n}^{\left(2\right)}\right)\sim \;\text{Uniform}\left(0,1\right)$ in two classes; Step 2 Initiate in two classes; Step 3 Set ${\text{u}}_{n}^{\left(1\right)*}={\text{u}}_{n}^{\left(1\right)}$, ${\text{u}}_{n}^{\left(2\right)*}={\text{u}}_{n}^{\left(2\right)}$, ${\text{y}}_{c_{1}}^{\left(1\right)*}={\text{y}}_{c_{1}}^{\left(1\right)}$, ${\text{y}}_{c_{2}}^{\left(2\right)*}={\text{y}}_{c_{2}}^{\left(2\right)}$; *iter* = 0; Do Step 4 Perform **Algorithm 1** on **X**_1_; ** Step 5** Perform **Algorithm 1** on **X**_2_; *iter* = *iter* + 1; Until **Y**^(1)*^ is |**Y**^(1)*^(ν) – **Y**^(1)*^(ν – 1)| ≤ ε or *iter* > *N_*iter*_*

The numbers of clustering in two classes are *C*_1_ and *C*_2_, and the cluster centers in two classes are **Y**_1_ and **Y**_2_, respectively. After applying Algorithm 2 on the whole data, the center matrix **Y** can be described by Y^*^ = [**Y**^(1)*^; **Y**^(2)*^].

### Consequent Parameter Learning in PCB-ICL-TSK

In this section, we compute the noise-insensitive consequent parameters in PCB-ICL-TSK. As discussed before, using the obtained the antecedent parameters, the dataset $\text{X}={\left\{{\text{x}}_{i},l_{i}\right\}}_{i=1}^{N}$ is represented as $S={\left\{\left(\tilde{\mu }\left({\text{x}}_{i}\right),l_{i}\right)\right\}}_{i=1}^{N}$, where $\tilde{\mu }\left({\text{x}}_{i}\right)={\left[{\tilde{\mu }}_{1}{\left({\text{x}}_{i}\right)}^{T},{\tilde{\mu }}_{2}{\left({\text{x}}_{i}\right)}^{T},\ldots ,{\tilde{\mu }}_{\left(C_{1}+C_{2}\right)}{\left({\text{x}}_{i}\right)}^{T}\right]}^{T}$. Defining the vector $d\left({\text{x}}_{i}\right)={\left[{\tilde{\mu }}_{1}{\left({\text{x}}_{i}\right)}^{T},{\tilde{\mu }}_{2}{\left({\text{x}}_{i}\right)}^{T},\ldots ,{\tilde{\mu }}_{\left(C_{1}+C_{2}\right)}{\left({\text{x}}_{i}\right)}^{T},1\right]}^{T}$, the consequent vector ${\text{p}}^{*}={\left[p_{0}^{1},p_{0}^{2},...,p_{0}^{{\;}_{\left(C_{1}+C_{2}\right)}},w\right]}^{T}$ can be computed by

(23) $$\begin{array}{l} f\left({\text{x}}_{i}\right)={\left({\text{p}}^{*}\right)}^{T}d\left({\text{x}}_{i}\right)={{\text{p}}_{0}}^{T}\tilde{\mu }\left({\text{x}}_{i}\right)+w\{\begin{array}{c} \geq 0,\;{\text{x}}_{i}\in {\text{X}}_{1}\\ <0,\;{\text{x}}_{i}\in {\text{X}}_{2} \end{array} \end{array}$$

where the vector ${\text{p}}_{0}={\left[p_{0}^{1},p_{0}^{2},...,p_{0}^{{\;}_{\left(C_{1}+C_{2}\right)}}\right]}^{T}$ and *w* is the decision threshold. If we multiply Equation (23) by the class label, Equation (23) is represented as *l*_*i*_(p^*^)^*T*^*d*(**x**_*i*_) ≥ 0 (*i* = 1, …, *N*). Then, the vector p^*^ can be computed by

(24) $$\begin{array}{l} l_{i}{\left({\text{p}}^{*}\right)}^{T}d\left({\text{x}}_{i}^{\;}\right)\geq \varepsilon_{0} \end{array}$$

In particular, ε_0_ = 1 leads to the classical SVM. For simplicity, we set ε_0_ = 1, and Equation (24) can be written as *l*_*i*_(p^*^)^*T*^*d*(**x**_*i*_) ≥ 1. Thus, Equation (24) can be written as

(25) $$J(p^{*})=\sum_{i=1}^{N}(l_{i}{\left(p^{*}\right)}^{T}d(x_{i})-1{)}^{2}$$

Denote the matrix **D** = [*l*_1_*d*(**x**_1_)^*T*^, *l*_2_*d*(**x**_2_)^*T*^, …, *l*_*N*_*d*(**x**_*N*_)^*T*^]^*T*^ and the error vector **e** = D^*^p^*^ – **1**. Equation (25) can be rewritten as

(26) $$\begin{array}{l} \underset{{\text{p}}^{*}}{\min}J\left({\text{p}}^{*}\right)=\frac{1}{2}{\left(\text{D}{\text{p}}^{*}-1\right)}^{T}\text{H}\left(\text{D}{\text{p}}^{*}-1\right) \end{array}$$

where the matrix **H** = (λ/*N*)diag(*h*_1_, *h*_2_, …, *h*_*N*_), with *h*_*i*_ = 0 for error *e*_*i*_ ≥ 0 and 1 otherwise.

However, the misclassification error in Equation (24) is noise sensitive. To further improve the robustness of the TSK fuzzy system, we use the asymmetric expectile term, which is noise insensitive, especially to noise around the decision boundary. The weight *h*_*i*_ of the *i*th sample can be expressed by

(27) $$\begin{array}{l} h_{i}=\{\begin{array}{c} q,\;e_{i}\geq 0\\ \left(1-q\right),\;e_{i}<0 \end{array} \end{array}$$

where *h*_*i*_ is the *q* (lower) expectile parameter. Obviously, when *q* = 0, the loss term obtained in Equation (27) is equal to the hinge loss, and when *q* = 0.5, the loss term is equal to the *l*_2_ loss in Huang et al. (2014a,b).

At the same time, considering the regularization term, Equation (26) can be rewritten as

(28) $$\begin{array}{l} \underset{{\text{p}}^{*}}{\min}J{\left({\text{p}}^{*}\right)}^{\left(k\right)}=\frac{1}{2}{\left({\text{D}}^{*}{\left({\text{p}}^{*}\right)}^{\left(k\right)}-1\right)}^{T}{\text{H}}^{\left(k\right)}\left({\text{D}}^{*}{\left({\text{p}}^{*}\right)}^{\left(k\right)}-1\right)\\ \;+\frac{\tau }{2}{\left({{\text{p}}_{0}}^{\left(k\right)}\right)}^{T}{{\text{p}}_{0}}^{\left(k\right)} \end{array}$$

where τ is the regularization parameter. p^*^^(k)^, **H**^(k)^, and **e**^(k)^ are the *k*th iteration of p^*^, **H**, and **e**, respectively.

The condition for optimality of Equation (28) in the *k*th iteration is obtained by setting *dJ*/*d*p^*^ = 0:

(29) $$\begin{array}{l} {\left({\text{p}}^{*}\right)}^{\left(k\right)}={\left({\left({\text{D}}^{*}\right)}^{T}{\text{H}}^{\left(k\right)}{\text{D}}^{*}+\tau \;\tilde{\text{I}}\right)}^{-1}{\left({\text{D}}^{*}\right)}^{T}{\text{H}}^{\left(k\right)}1 \end{array}$$

where $\tilde{\text{I}}$ is the identity matrix with the last element on the main diagonal set to 0.

The consequent parameter learning in IB-TSK-FC on dataset **X** is shown in Algorithm 3.

**Algorithm 3:** Learning algorithm for consequent parameters.

**Input:** The dataset **X**; the number of clusters (*C*_1_ + *C*_2_); the cluster centers **Y**_1_, **Y**_2_ and the membership matrix **U**_1_, **U**_2_; the expectile parameter *q*; and the regularization parameter τ;
**Output:** Consequent parameters **p**_0_.
Step 1 Run **Algorithm 2** to obtain the antecedent parameters; Step 2 Compute the membership function *d*(**x**_*i*_)=${\left[{\tilde{\mu }}_{1}{\left({\text{x}}_{i}\right)}^{T},{\tilde{\mu }}_{2}{\left({\text{x}}_{i}\right)}^{T},\ldots ,{\tilde{\mu }}_{\left(C_{1}+C_{2}\right)}{\left({\text{x}}_{i}\right)}^{T},1\right]}^{T}$ by using Equations (5)–(7); *k* = 0; Do Step 3 Obtain the parameters (p*)^(k)^ using Equation (29); Step 4 Compute the parameter **e**^(k)^ using **e**^(k)^ = **D**(p*)^(k)^ – **1**; Step 5 Compute the parameter **H**^(k+1)^ using Equation (27); *k* = *k* + 1; Until p* is convergence or *k* > *k*_max_.

## Experiment

### Experimental Settings

The real-world EEG signals have characters of high dimensionality and instability. Feature extraction is a necessary stage before classification for EEG signal recognition. In general, time domain and frequency domain feature extractions are two types of feature extraction methods (Wen and Zhang, 2017). In our experiments, we extract EEG features using kernel principal component analysis (KPCA) and short-time Fourier transform (STFT) (Blanco et al., 1997). The former is the time domain feature extraction, and the latter is the frequency domain feature extraction. In the experiment, we design eight classification tasks, namely, four binary classification and four three-class classification tasks, as shown in Table 2. We corrupt the original datasets with different amounts of random noises at 5, 10, and 15% noise levels.

**TABLE 2:** EEG classification tasks in the experiment.

**Tasks**	**Number of classes**	**Datasets**
T1	Two classes	A and C
T2		A and E
T3		B and D
T4		B and E
T5	Three classes	A, C and E
T6		A, D, and E
T7		B, C, and E
T8		B, D, and E

The experimental environment in this study is a computer with Intel Core i3-3317U 3.40-GHz CPU and 8-GB RAM. To validate the performance of MST-TSK, we compare three fuzzy systems (FS-FCSVM; et al., 2007, ε-margin-TSK-FS; Leski, 2005, and IB-TSK-FC; Gu et al., 2017b) and two robust classification methods (CS-SVM; Iranmehr et al., 2019 and FRSVM-ANCH; Gu et al., 2019). The Gaussian kernel is used for two SVM methods. The parameter settings for all methods are listed in Table 3. All parameters are obtained by a 5-fold cross-validation strategy.

**TABLE 3:** Parameter settings for all methods in the experiment.

**Methods**	**Parameter settings**
ε-margin-TSK-FS FS-FCSVM	Number of rules ∈ {1, 2, …, 12}, regularization parameter ∈ {10^−3^, 10^−2^, …, …, 10^3^}, scale parameter ∈ {0.4^2^, 0.6^2^, …, 3^2^}, fuzzy index = 2
IB-TSK-FC	Model sparsity parameter ∈ {1, 2, …, 6}, fuzzy index = 2, number of particles = 10, convergence thresholds = 10^−3^, convergence threshold = 10–^3^, strength parameter = 3
CS-SVM	Gaussian kernel parameter ∈ {10^−2^, 10^−1^, …, …, 10^2^}, regularization parameter ∈ {10^−3^, 10^−2^, …, …, 10^3^}
FRSVM-ANCH	Gaussian kernel parameter ∈ {10^−2^, 10^−1^, …, …, 10^2^}, regularization parameter ∈ {10^−3^, 10^−2^, …, …, 10^3^}, pinball loss parameter = 0.05
PCB-ICL-TSK	Fuzzy index = 2, number of rules ∈ {1, 2, …, 12}, convergence threshold = 10^−3^, strength parameter = 3, expectile parameter = 0.05, maximum number of iterations = 1,000 and 200 in Algorithms 1 and 2, respectively

### Classification Performance Comparison

In this section, eight EEG classification tasks are used to verify the classification performance of PCB-ICL-TSK. Tables 4, 5 show the experimental results of six classification algorithms using STFT and KPCA feature extraction methods at the 5% noise level. Tables 6, 7 show the experimental results of six classification methods using STFT and KPCA feature extraction methods at the 10% noise level. Tables 8, 9 show the experimental results of six classification methods using STFT and KPCA feature extraction methods at the 15% noise level. From the experimental results, it can be seen that the noise data seriously affect the classification performance of the method. During the learning process, considering the noise of the data is helpful to promote the classification performance. Therefore, the performances of FS-FCSVM, ε-margin-TSK-FS, and IB-TSK-FC are poor. CS-SVM, FRSVM-ANCH, and PCB-ICL-TSK are not sensitive to noise, and they can achieve good classification results. In particular, PCB-ICL-TSK shows excellent classification performance in different levels of noise occasions, and it reflects strong robustness. Since PCB-ICL-TSK uses the PCB-ICL and Ho–Kashyap procedure with an asymmetric expectile term to compute antecedent and consequent parameters of fuzzy rules, it is noise insensitive. In addition, in the Bayesian framework, PCB-ICL obtains global optimal clustering results, and the strategy of competitive relationship of clustering centers can enhance the interpretability of the antecedents of fuzzy rules.

**TABLE 4:** The classification accuracy for the 5% noise level using STFT features.

**Tasks**	**FS-FCSVM**	**ε-margin-TSK-FS**	**IB-TSK-FC**	**CS-SVM**	**FRSVM-ANCH**	**PCB-ICL-TSK**
Task 1	94.56	94.69	95.22	96.27	96.73	**96.74**
Task 2	94.88	94.81	95.34	96.19	96.51	**96.62**
Task 3	94.64	94.57	95.39	96.45	**96.70**	96.69
Task 4	94.21	94.34	95.57	96.58	96.54	**96.67**
Task 5	93.46	93.25	93.98	96.27	**96.32**	96.29
Task 6	93.37	93.42	93.96	95.89	**96.14**	96.12
Task 7	93.25	93.34	93.86	95.78	95.97	**96.04**
Task 8	93.38	93.29	93.75	95.86	96.09	**96.10**

*The bold values indicate the best classification performance in the tasks*.

**TABLE 5:** The classification accuracy for the 5% noise level using KPCA features.

**Tasks**	**FS-FCSVM**	**ε-margin-TSK-FS**	**IB-TSK-FC**	**CS-SVM**	**FRSVM-ANCH**	**PCB-ICL-TSK**
Task 1	94.53	94.70	95.23	96.28	96.63	**96.75**
Task 2	94.92	94.78	95.39	96.23	96.50	**96.59**
Task 3	94.62	94.55	95.40	96.42	**96.72**	96.72
Task 4	94.23	94.33	95.56	96.62	96.51	**96.65**
Task 5	93.47	93.25	93.95	96.26	**96.33**	96.31
Task 6	93.38	93.44	93.97	95.94	96.11	**96.19**
Task 7	93.23	93.36	93.85	95.79	95.95	**96.01**
Task 8	93.37	93.34	93.76	95.89	96.10	**96.11**

*The bold values indicate the best classification performance in the tasks*.

**TABLE 6:** The classification accuracy for the 10% noise level using STFT features.

**Tasks**	**FS-FCSVM**	**ε-margin-TSK-FS**	**IB-TSK-FC**	**CS-SVM**	**FRSVM-ANCH**	**PCB-ICL-TSK**
Task 1	92.79	92.84	93.44	94.64	95.09	**95.29**
Task 2	93.12	93.01	93.58	94.57	95.21	**95.28**
Task 3	92.76	92.76	93.59	94.86	**95.39**	95.30
Task 4	92.48	92.52	93.76	95.03	94.99	**95.21**
Task 5	91.60	91.45	92.16	94.63	94.66	**94.93**
Task 6	91.61	91.65	92.23	94.23	**94.71**	94.70
Task 7	91.44	91.58	92.09	94.18	94.61	**94.68**
Task 8	91.58	91.55	91.94	94.19	94.56	**94.67**

*The bold values indicate the best classification performance in the tasks*.

**TABLE 7:** The classification accuracy for the 10% noise level using KPCA features.

**Tasks**	**FS-FCSVM**	**ε-margin-TSK-FS**	**IB-TSK-FC**	**CS-SVM**	**FRSVM-ANCH**	**PCB-ICL-TSK**
Task 1	92.75	92.80	93.39	94.61	95.24	**95.27**
Task 2	93.12	92.98	93.53	94.56	**95.21**	**95.21**
Task 3	92.77	92.72	93.63	94.85	95.14	**95.26**
Task 4	92.49	92.58	93.84	95.08	94.95	**95.20**
Task 5	91.63	91.42	92.20	94.66	94.77	**94.96**
Task 6	91.53	91.66	92.22	94.24	94.58	**94.69**
Task 7	91.45	91.52	92.10	94.24	94.51	**94.68**
Task 8	91.61	91.58	91.87	94.19	94.54	**94.69**

*The bold values indicate the best classification performance in the tasks*.

**TABLE 8:** The classification accuracy for the 15% noise level using STFT features.

**Tasks**	**FS-FCSVM**	**ε-margin-TSK-FS**	**IB-TSK-FC**	**CS-SVM**	**FRSVM-ANCH**	**PCB-ICL-TSK**
Task 1	90.73	90.77	91.39	92.98	93.48	**93.88**
Task 2	91.13	90.94	91.49	92.84	93.85	**93.90**
Task 3	90.70	90.76	91.53	93.18	93.70	**93.88**
Task 4	90.48	90.57	91.84	93.33	**93.85**	93.84
Task 5	89.68	89.39	90.19	92.91	93.07	**93.57**
Task 6	89.55	89.74	90.20	92.59	92.92	**93.33**
Task 7	89.49	89.48	90.05	92.48	93.04	**93.28**
Task 8	89.62	89.67	89.85	92.50	93.28	**93.32**

*The bold values indicate the best classification performance in the tasks*.

**TABLE 9:** The classification accuracy for the 15% noise level using KPCA features.

**Tasks**	**FS-FCSVM**	**ε-margin-TSK-FS**	**IB-TSK-FC**	**CS-SVM**	**FRSVM-ANCH**	**PCB-ICL-TSK**
Task 1	90.73	90.77	91.39	92.96	93.47	**93.87**
Task 2	91.13	90.94	91.49	92.86	93.34	**93.82**
Task 3	90.70	90.76	91.53	93.20	93.73	**93.90**
Task 4	90.48	90.57	91.84	93.33	**93.82**	**93.82**
Task 5	89.68	89.39	90.19	92.93	93.12	**93.49**
Task 6	89.55	89.74	90.20	92.52	92.91	**93.32**
Task 7	89.49	89.48	90.05	92.47	92.99	**93.24**
Task 8	89.62	89.67	89.85	92.49	93.20	**93.22**

*The bold values indicate the best classification performance in the tasks*.

### Interpretability Comparison

**Figure 3:**
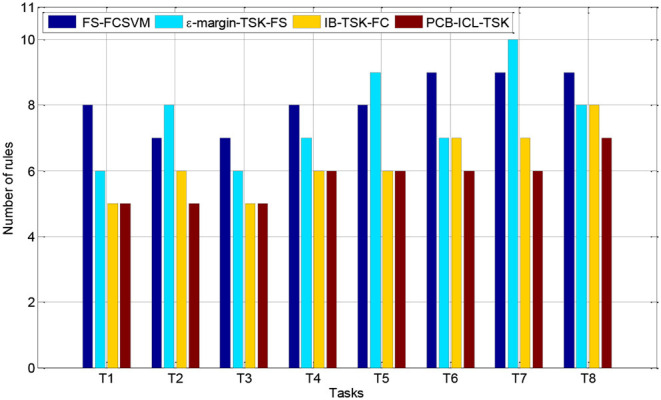
The rules obtained by four fuzzy systems on the 5% noise level using KPCA features.

**Figure 4:**
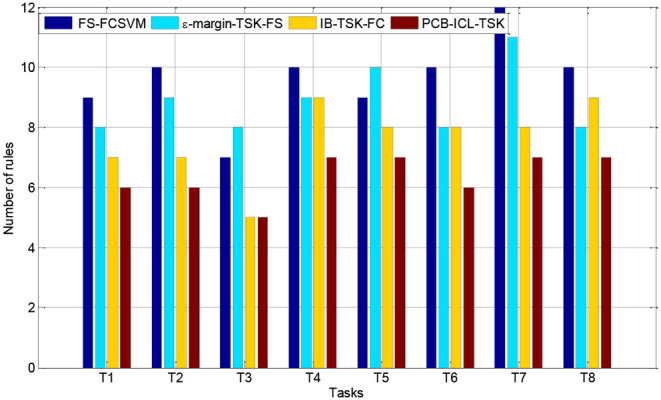
The rules obtained by four fuzzy systems on the 15% noise level using KPCA features.

**Figure 5:**
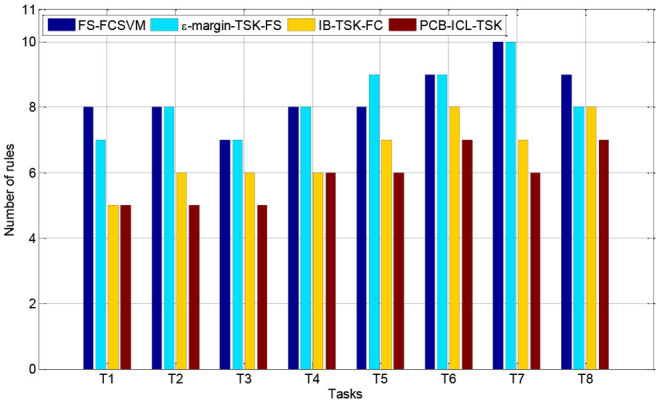
The rules obtained by four fuzzy systems on the 5% noise level using STFT features.

**Figure 6:**
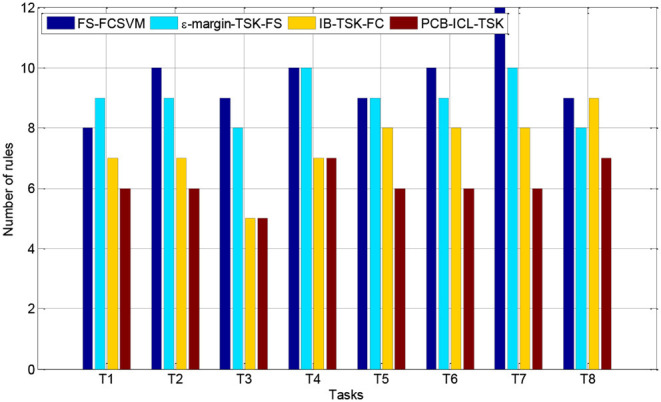
The rules obtained by four fuzzy systems on the 15% noise level using STFT features.

**Figure 7:**
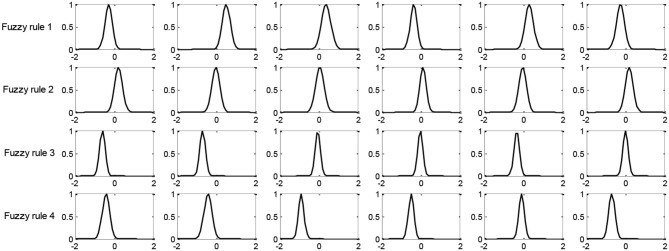
Fuzzy membership functions obtained by PCB-ICL on Task 1 with the 5% noise level using KPCA features.

In this section, we compare the number of fuzzy rules of four fuzzy systems in Task 8. Figures 3, 4 show the number of fuzzy rules on the 5 and 15% noise levels for four fuzzy systems using KPCA features. Figures 5, 6 show the number of fuzzy rules on the 5 and 15% noise levels for four fuzzy systems using STFT features. From the results in Figures 3–6, compared with the three fuzzy systems, the number of fuzzy rules obtained by PCB-ICL-TSK is the least in all EEG classification tasks. It is known that for fuzzy systems, the interpretability of fuzzy rules is related to the number of fuzzy rules and the definition of fuzzy subsets. The fuzzy membership function obtained by PCB-ICL on Task 1 at the 5% noise level using KPCA features is shown in Figure 7. Because PCB-ICL clustering considers the influence of clustering centers of different classes in the process of clustering, that is, the competition relationship between different classes of clustering centers, PCB-ICL clustering can obtain clustering centers with a large interval, which guarantees the partition clarity of feature space and the classification accuracy of the obtained fuzzy system and the interpretation of rules.

## Conclusion

The noise-insensitive PCB-ICL-TSK fuzzy system is proposed in this paper. In the learning of rule antecedent parameters, the proposed noise-insensitive PCB-ICL clustering based on the Bayesian probability model is used. PCB-ICL clustering considers the repulsion between different clustering centers, which can ensure the interpretability of the rule antecedent. PCB-ICL can learn the global optimal solution of clustering results by using the Markov model. PCB-ICL-TSK learns consequent parameters using the Ho–Kashyap procedure with an asymmetric expectile term. Thus, it not only has strong noise resistance but also has high classification performance. The experimental results of a real EEG dataset show that PCB-ICL-TSK has achieved satisfactory results in classification performance and high interpretability. Our future work is to further improve its practicability when the sample dimension is large.

## Data Availability Statement

Publicly available datasets were analyzed in this study. This data can be found here: The dataset analyzed for this study can be found in the Department of Epileptology University of Bonn [http://epileptologie-bonn.de/cms/upload/workgroup/lehnertz/eegdata.html].

## Author Contributions

TN and XG conceived and developed the theoretical framework of the manuscript. All authors carried out the experiment and data process and drafted the manuscript.

## Conflict of Interest

The authors declare that the research was conducted in the absence of any commercial or financial relationships that could be construed as a potential conflict of interest.
